# Pharmacovigilance: Boon for the safety and efficacy of Ayuvedic formulations

**DOI:** 10.4103/0975-9476.74427

**Published:** 2010

**Authors:** Anand Chaudhary, Neetu Singh, Neeraj Kumar

**Affiliations:** 1 Regional Pharmacovigilance Centre for Ayurvedic Drugs, North Zone; 2 Department of Rasa Shastra, Institute of Medical Sciences, Banaras Hindu University, Varanasi, India

**Keywords:** Adverse drug reaction, Awareness, Ayurvedic medicine, Pharmacovigilance, Safety

## Abstract

Pharmacovigilance is a corrective process originating in pharmaco-epidemiology. The 1997 Erice Declaration, presented at the World Health Organisation, became the basis on which the concept was implemented internationally for conventional systems of medicine. The increasing international acceptance of Ayurveda, led regulators to implement a similar program for Ayurveda, particularly as some medical professionals, scientists and members of the public reported adverse reactions after taking Ayurvedic formulations. The World Health Organisation therefore persuaded the Department of AYUSH, Ministry of Health and Family Welfare, Government of India, to implement a pharmacovigilance program for Ayurveda, as a means to ensuring the safety and efficacy of Ayurvedic medicines. After a year of due diligence, the pharmacovigilance program was launched nationally on 29 September 2008. Since that time, Ayurveda, Siddha and Unani medicines have been monitored according to the provisions of a protocol prepared by the National Pharmacovigilance Resource Centre, IPGTRA, Jamnagar, and approved by Department of AYUSH. The program was reviewed, first, on 21st January 2009 by the National Pharmaco-vigilance Consultative Committee for ASU drugs (NPCC-ASU), and again, on 15 Feburary, 2010, when an evaluation meeting effectively rubber stamped the program. Among the outcomes of these meetings were several suggestions of measures to improve the program’s efficiency. Recent developments include the constitution of pharmacovigilance centers at all Ayurveda Teaching institutes and research centers.

## INTRODUCTION

The 1980’s were witness to the 20^th^ century’s third upsurge in Ayurveda’s popularity with westerners. The first two, in the 1920’s and 1940’s, failed to sustain on account of the discovery of antibiotics like the sulfa drugs and penicillin, but once again due to lack of cures for chronic disease and side-effects of western drugs, developed countries started looking to Ayurveda for treatments to restore wellness.[1]

As a medical science, Ayurveda includes considerations of the natural human life-span and life-cycle, awareness of the nature of human life on earth, and reasons for it. True integration of body, mind and spirit, of the psychic and the somatic, are among its unique features. Being a system of preventive medicine and health promotion, its accurate application results in a full life-span enjoyed in healthy well-being.[2]

In its origins Ayurveda was carefully and systematically developed. As a result, it is now being confirmed by measures of many scientific parameters. It not only provides well-based medical cures for disease, but its holistic approaches use unique principles of diet, life-style and, particularly, therapeutics, to balance and enrich all aspects of the physiology and psyche. In light of these aspects of Ayurveda past and present, the system’s potential for promotion of health and wellness is well-acknowledged.[3]

On the other side of the coin, Ayurveda treatments have come under attack for several reasons. Unethical companies are under scrutiny for the production of adulterated and misbranded medicaments by inaccurate methods, while some of its practitioners have indulged in illegal practice.[3]

All these events led the Department of AYUSH, Ministry of Health and Family Welfare, Government of India, to implement a National Pharmacovigilance Program for Ayurveda, Unani, and Siddha systems of medicine, in order to systematically monitor adverse drug reactions (ADR).[4]

## PHARMACOVIGILANCE IN AYURVEDA

Pharmacovigilance is defined as ‘the detection, assessment, understanding, and prevention of adverse effects of drugs or any other possible drug related problems’. This definition plainly covers the objectives of the AYUSH program and its coverage area as per the WHO guidelines.[5]

Although a technical term equivalent to “pharmacovigilance” does not feature in Ayurvedic texts, the spirit of pharmacovigilance is vibrant throughout Ayurveda’s classical literature. The Brihattrayi and Laghutrayi repeatedly emphasize the major goals of pharmacovigilance, to improve patient care and safety during treatment, and thus to promote rational use of medications. These are recurrent themes of Ayurvedic pharmacology (*Dravyaguna*), pharmaceutics (*Rasa Shastra and Bhaishjya Kalpana*), and therapeutics (*Chikitsa*).[6] It is probable that these basic principles of Ayurveda gave rise to the common belief that Ayurvedic medicines are safe.

The Ayurvedic literature gives details of drug-drug and drug-diet incompatibilities based on elaborately described qualitative differences in ingredients or quantitative proportions. These factors undoubtedly prevent the onset of many otherwise unfortunate reactions. Ayurveda’s *Anupan* therapeutic method and *Shodhan* pharmaceutics principles probably also contribute to the prevention of many undesired and unforeseen events. Prevention of this kind is a major goal of pharmacovigilance programs.

## PHARMACOVIGILANCE PROGRAM CHRONOLOGY

### Conventional medicine

According to Article 2 of the World Health Organization’s constitution, its mandate from its Member States[5] is “to develop, establish, and promote international standards with respect to food, biological, pharmaceutical and similar products.” Similarly, Article 21 mandates that it adopt regulations concerning “standards with respect to the safety, purity and potency of biological, pharmaceutical and similar products moving in international commerce.”

In accordance with these resolutions, WHO has acted very quickly whenever widespread health disorders have arisen. The 1961Thalidomide disaster was the driving stroke which compelled WHO to initiate a program to thoroughly monitor all prescribed drugs. In its sixteenth World Health Assembly in 1963, resolution WHA 16:36 emphasized the need for rapid dissemination of information on ADRs.[5]

In 1968 as a result of reports of drugs producing adverse reactions, WHO initiated its *Pilot Research Project for International Drug Monitoring*. The program fulfilled basic purposes.[5] Finally, in 1997, WHO drew up the *Erice Declaration*, an international agreement eventually signed by all member states to agree on uniform standards for reporting ADR’s.

## THE ERICE DECLARATION

The Erice Declaration represented significant progress. It challenges all players, including:

- Public Health administrators
- Health professionals
- The Pharmaceutical industry
- Governments
- Drug regulators
- Media, and
- Consumers

“to strive towards the highest ethical, professional, and scientific standards in protecting and promoting the safe use of medicines.” The declaration charges governments and all involved in determining policies relating to the benefit, harm, effectiveness, and risk of medicines, to be accountable for what they communicate to both the public and patients. It calls for honesty when communicating drug safety information, even when such information is incomplete and investigations are still underway. It further stipulates that patients be transparently informed of all facts, assumptions, and uncertainties concerning safety profiles of their medicines. Considerable effort has subsequently been made to achieve its goals.[7]

Further in 2002, India agreed to send all its ADRs arising from use of conventional medicine to WHO’s ADR Monitoring Centre in Uppsala, Sweden, where its international ADR database receives reports from National Centers in 65 countries using ‘MedDRA’ and WHO ART terminology: the ‘Medical Dictionary for Drug Regulatory Activities’ and ‘World Health Organization Adverse Reaction Terminology’ for ADR’s.[8]

India introduced the program in accordance with obligations from agreements signed in 1997 and 2002. A new national network of pharmacovigilance centers was established in 2004, coordinated by the Central Drugs Standard Control Organisation (CDSCO).[9] Previously, from 1980, the program was coordinated by ICMR (Indian Council of Medical Research) through its adverse drug reporting centers,[7] up to 1986, when a formal ADR monitoring system was introduced. This consisted of 12 regional centers, each responsible for a population of 50 million.[10] In this program, it is compulsory to report any ADR from a physician, and as an essential component of drug safety, pharmaceutical companies must also submit periodic safety update reports based on postmarketing surveillance.[11]

A common understanding between all the pharmacovigilance program’s conventional medicine stakeholders in India was that, to deal with certain objectives not addressed by the 2004 system, the program should be reorganized. The program was relaunched in April 2010 by CDSCO in association with a new key player, the Department of Pharmacology of the All India Institute of Medical Sciences, New Delhi. Up to now, more than 40 medical colleges have been nominated as pharmacovigilance centers under a policy aiming to cover all medical colleges in the country. For conventional medicine, the program has thus been redesigned, and implemented in the hope that it will provide a fool-proof system of ensuring medicines’ safety.[12,13]

## AYURVEDIC MEDICINE

The idea of a corresponding pharmacovigilance program for traditional medicine[14] began in November 2006, principally led by the Department of Clinical Pharmacology, TNMC and BYL Nair Ch Hospital, Mumbai. In collaboration with WHO, clinical pharmacologist Urmilla Thatte and Vaidya Supriya Bhalerao organized a workshop, “Pharmacovigilance of Ayurvedic Medicine” on 20 and 21 November, 2006.[15]

In October, 2007, BHU’s Institute of Medical Sciences’ Department of Rasa Shastra organized a seminar-cum-workshop entitled “Safety Profile of Ayurvedic Dosage Forms”. The seminar’s technical report submitted to WHO strongly influenced how the Ayurvedic medicines’ pharmacovigilance safety program was implemented.[16]

The next concrete step sponsored by WHO was taken by the Institute of Post Graduate Teaching and Research in Ayurveda (IPGTRA), at Gujarat Ayurveda University, Jamnagar, which organized a workshop on the possibility of implementing pharmacovigilance programs for Ayurvedic medicine in December, 2007. In view of the program’s potential importance, the Dept of AYUSH requested IPGTRA to prepare a protocol and ADR reporting format to implement pharmacovigilance for Ayurveda, Siddha, and Unani (ASU) drugs.[17]

The draft protocol was discussed and technically analyzed by a consultative committee of concerned experts consisting of specialists in different fields (i.e. pharmacologists and Vaidyas in different disciplines) in Delhi in August, 2008, sponsored by the WHO national office. Incorporating expert suggestions, the draft was finalized and released by Department of AYUSH Secretary, Ms Anita Das, on 29 September 2008. IPGTRA was subsequently declared National Pharmacovigilance Resource Centre for ASU Drugs. From that date, India’s present ASU systems pharmacovigilance program has been in operation.[17] The structure of its recommended procedures was made at a meeting group of experts in August, 2008, and is given in Box 1. For precise definitions of terms, Table 1.

**Table 1:** Terminology of pharmacovigilance for Ayurveda, Siddha and Unani

**Side effect:** Any unintended effect of a pharmaceutical product occurring at doses normally used in man which is related to the pharmacological properties of the drug.
**Adverse event / Adverse experience:** any untoward medical occurrence that may present during treatment with a pharmaceutical product, but which does not necessarily have a causal relationship with the treatment.
**Signal:** Reported information on a possible causal relationship between an adverse event and a drug, the relationship being previously unknown or incompletely documented. Usually more than a single report is required to generate a signal, depending on the seriousness of the event, and the quality of the information.
**Adverse reaction:** A response to a drug which is noxious and unintended, and which occurs at doses normally used in man for the prophylaxis, diagnosis, or therapy of disease or modification of physiological function.

A glossary of basic pharmacovigilance terms. Other definitive terms used in reporting ADRs, are beyond the scope of this article.

**Box 1**

Reporting procedures for India’s pharmacovigilance program for ASU drugs

**The structure of India’s pharmacovigilance program for ASU drugs**

India’s National Program of Pharmacovigilance for ASU drugs was adopted under the following blueprint in accordance with recommendations of the expert group made at its meeting on 28-29 August, 2008.

**What to report**

The National Pharmacovigilance Programme for ASU drugs (NPP ASU) shall encourage reporting of all suspected drug related adverse events, including those suspected to have been caused by interaction with any other drugs or food incompatibilities. Reporting of seemingly insignificant or common adverse reactions may be important, since it could highlight a widespread prescribing problem.

The program particularly solicits reports of

- all adverse reactions suspected to have been caused by ASU drugs either alone or in conjunction with other drugs
- all suspected drug interactions
- reactions to any other drugs suspected of significantly affecting a patient’s management, including reactions suspected for events in the following categories
  - death
  - life threatening (real risk of dying)
  - hospitalization (initial or prolonged)
  - disability (significant, persistent, or permanent)
  - congenital anomaly
  - required intervention to prevent permanent impairment or damage.

The prescribed ‘Adverse Drug Event Reporting Form for ASU Drugs’ shall be used for the purpose of the National Pharmacovigilance Programme for ASU. The form may be downloaded from the webpages of Gujarat Ayurveda University or WHO’s India Office.[19,20]

**Who can report**

Any health care professional may report suspected adverse drug events. The program does not accept reports from lay members of the public, nor others than health care professionals. Others can report through the physician under whom they have undergone treatment.

**Where to report**

Reporting should be done in a prescribed format through a local pharmacovigilance center.

**Direction of submitted information**

Information in the forms is to be handled in all confidentiality. Peripheral pharmacovigilance centers forward the form to their regional pharmacovigilance centers where causality analysis is carried out. The information is then forwarded to the National Pharmacovigilance Resource Centre, where it is consolidated, statistically analyzed, and forwarded to the Dept of AYUSH.

## SET UP OF PHARMACOVIGILANCE FOR ASU DRUGS

Departments of government, hospitals, and academic institutions, involved in clinical pharmacology, clinical pharmacy, clinical toxicology, or epidemiology have been identified as the best hosts to set up and house centers. Those applying will be assessed for infrastructure and other resource requirements, since proper planning is essential to establish and run a pharmacovigilance center successfully. Also, government support is necessary at least at the national level.

At present, besides the National Center in Jamnagar, 8 Regional Centers and 30 Peripheral Centers (with an aim to open pharmacovigilance are being developed to carry out program activities such as receiving reports submitted through the www.ayurveduniversity.com portal, where a complete list of centers may be found. Continuing Medical Education and public meetings are being conducted to raise health professional’s awareness of ASU drugs.

## SCOPE OF PHARMACOVIGILANCE IN AYURVEDA

The goals of Ayurveda’s pharmacovigilance program are to improve:

- Patient care and safety when using Ayurvedic medicines and related interventions;
- Public health and safety records of Ayurvedic medicines
- Assessment of benefit, harm, effectiveness, and risk of medicines,
- Encouragement of safe, rational, and more effective (including cost effective) use, and promotion of understanding, education, and clinical training in pharmacovigilance for Ayurvedic medicines and its effective communication to the public. Many cases have been reported in the recent past regarding ADRs and drug-drug interactions at various national and international forums.[21]

## DISCUSSION

That Ayurvedic drugs are safe due to their natural origin is a popular conception, but it is true to the extent that many are used as foodstuffs and deserve the classification Generally Recognized as Safe - GRAS. References in the Ayurvedic classics to possible adverse reactions to certain Ayurvedic medicines alert us against accepting this concept as universal, however. This is particularly true if medicines are not prepared properly, or if preconditions for their administration are not respected by both physician and patient, which will increase the possibility of an ADR occurring. Charak Sutra Sthana 26 warns that substances contrary to *deha dhatus* will be antagonistic (*virodha*) towards them. Such antagonism may result from properties, combination, processing, place, time, dose etc. or natural composition.

In Ayurveda, diet (*ahar*) is as important for cure as medicine. The texts state that an ailment can only be cured by following proper diet (*pathya*). In this context, Charaka lists dietary incompatibilities between particular foodstuffs[22]:

- Fish and milk
- Honey and ghee in equal quantity
- Hot water after taking *bhallataka*
- *Kampillaka* cooked with butter milk.

### Antagonistic food may cause ADRs

Ayurveda’s overall approach is holistic in that it aims to return a patient’s physiology to its natural state of balance. Effects of Ayurvedic drugs are considered in terms of their ability to return the physiology to its natural, holistic state. Improper dose schedules either in quantity or duration may lead to ADRs. Similarly, for any herbal or herbo-mineral formulations used in overdose or for excessive time periods.

The pharmacovigilance program promises to close the gap between Ayurvedic drugs’ potential and reality. Some Ayurvedic citations use flowery language to describe certain formulations’ therapeutic value, for example, the effect of *Grahani Kapat Rasa* on dysentery / diarrhea; similarly for certain *vajikarna* formulations. There may be a huge gap between claimed and reality, suggesting assessment of their genuine value under pharmacovigilance.

An important aspect of the pharmacovigilance program is formulations’ economic evaluation, which may be carried out at any stage in a health care strategy’s life cycle. Data from such studies differs from that for clinical trials, and requires business-style analysis. Such economic evaluation studies must be considered an integral component of a decision analysis system using multiple criteria and methodologies from different disciplines, that helps the program achieve its multifaceted targets.

When new drugs are developed, the pharmacovigilance program with its social perspective requires economic evaluation of all aspects of their use in treatment, including side effects, adverse reactions, and their additional treatment costs, in addition to routine therapeutic evaluation. The pharmaceutical industry also needs to take responsibility for these added facets of pharmacovigilance.

The program may also be applied in cases where medicines are unavailable, unaffordable, unsafe, or improperly used; or where conflict of interest with manufacturers occurs, or in poorly monitored clinical trials, when patient recruitment is unethical or informed consent is inadequate. According to the December, 2008 notification of Rule 170 of the Drugs and Cosmetics Rule, 1945, many clinical trials are planned in future.[23]

Successful implementation of the pharmacovigilance program, requires pharmaceutical companies to demonstrate to both regulators and consumers that they are doing everything possible to assure drug safety, including developing more effective ways to manage drug safety data. Efficient analysis of data from adverse event reporting systems, and internal and external data sources are needed to respond to regulators’ safety inquiries or other issues.[24]

The program was reviewed on 21 January 2009 by the National Pharmaco-vigilance Consultative Committee for ASU drugs (NPCC-ASU). More recently, on 15 February, 2010, the Secretary, Department of AYUSH chaired an evaluation meeting of the Pharmacovigilance Program for ASU Drugs, new initiatives to implement the program more effectively. The evaluation meeting effectively rubber stamped the program. Among the outcomes of these meetings were several suggestions of measures to improve the program’s efficiency. NPRC, Jamnagar, presented details of 103 ADRs reported from all over the country and their causality assessment. This is a matter of some concern. More recent developments include constitution of pharmacovigilance centers at all Ayurveda teaching institutes and research centers.

## CONCLUSION

On the basis of the above, we conclude that there is a need for a proper postmarketing surveillance program to observe quality, safety, and efficacy of Ayurvedic drugs, which is now available in the form of National Pharmacovigilance Programme for ASU Drugs.

The success of any pharmacovigilance system lies in its ability to prevent further adverse reactions on the basis of information received. This will be possible only when physicians are vitally alert to the onset or offset of any ADRs. They need to prioritize their contributions to make the pharmacovigilance program for Ayurvedic medicines a success.
